# Deep learning-based infrared thermography reveals reproducible uniform and individual thermoregulatory responses during running

**DOI:** 10.1038/s41598-026-44102-6

**Published:** 2026-03-28

**Authors:** Vincent Weber, Daniel Andrés López, David Tobias Ochmann, Severin Zentgraf, Markus Nägele, Elmo W. I. Neuberger, Elmar Schömer, Perikles Simon, Barlo Hillen

**Affiliations:** 1 Department of Sports Medicine, Disease Prevention and Rehabilitation, Institute of Sports Science, Faculty of Social Science, Media and Sports, Johannes Gutenberg University, Mainz, Germany; 2 Institute of Computer Science, Research Group Computational Geometry, Johannes Gutenberg University, Mainz, Germany; 3 Optoprecision GmbH, Bremen, Germany; 4 Institute of Occupational, Social, and Environmental Medicine, University Medical Center of the Johannes Gutenberg University Mainz, Mainz, Germany

**Keywords:** Infrared thermal imaging, Thermography, Core body temperature, Reproducibility, Exercise physiology, Deep neural network, Artificial intelligence, Medical research, Physiology

## Abstract

Infrared thermography (IRT) has recently gained attention in the field of exercise physiology, due to its ability to monitor thermoregulatory and cardiopulmonary responses non-invasively and in real time during physical exercise. However, the reproducibility of intra-individual measurement and standardization of region-of-interest selection in relation to the acute exercise response remain inconclusive. This study aimed to examine the reproducibility and physiological relevance of specific skin temperature (T_SK_) metrics processed automatically using deep learning-assisted IRT during running, and to synchronize these metrics with cardiopulmonary and thermoregulatory parameters. Eleven endurance-trained individuals performed three 46-min running sessions over 2 days, with the same average external load but different intensity distributions. Individual anaerobic threshold velocity (vIAT), previously determined by cardiopulmonary exercise testing, was used to prescribe running intensity. During exercise, oxygen consumption (VO_2_), core temperature (T_CORE_), heart rate (HR) and different T_SK_ metrics, including non-vessel (T_NV_), cutaneous arterial perforator (T_P_), and superficial vein patterns, were continuously measured. All T_SK_ metrics displayed consistent temporal dynamics aligned with external load, but their absolute temperature levels differed systematically. During intermittent running and recovery, T_P_ exhibited robust correlations with HR and VO_2_ (*r* = − 0.63 to − 0.9, *p* < 0.001), and T_P_ entropy showed consistent associations with T_CORE_ during the warm-up (*r* = 0.59–0.83, *p* < 0.001). This indicates uniform response patterns across the cohort. In contrast, T_NV_ demonstrated heterogeneous correlations with T_CORE_, depending on individual exercise capacity. A strong inverse correlation was identified between ∆T_NV_ and vIAT (*r* = − 0.74 to − 0.88, *p* ≤ 0.009) and individuals with higher vIAT demonstrated greater T_CORE_-T_NV_ gradients during running. Measurements of ∆T_NV_ demonstrated high reproducibility, with intra-individual ICC(3,1) values of 0.89 for recovery and 0.76 for warm-up, and no statistically significant differences between the three sessions. Deep learning-assisted IRT provides reproducible, physiologically consistent metrics across repeated exercise sessions, regardless of the day or prior load. Distinct T_SK_ metrics capture both uniform and individual-specific thermoregulatory responses. Variability in peripheral temperature regulation is more strongly associated with running velocity at the individual anaerobic threshold than with maximal cardiorespiratory fitness.

**Supplementary Information:**

The online version contains supplementary material available at 10.1038/s41598-026-44102-6.

## Introduction

The application of infrared thermography (IRT) during physical exercise is promising because it enables non-contact detection of acute cardiovascular and thermoregulatory responses^1,2^ based on exercise-induced variations in skin temperature (T_SK_)^3^. Furthermore, given the interplay between core temperature (T_CORE_) and T_SK_, for example, through vascular reactivity, T_SK_ measurements can support diagnostics in cardiovascular health^4^, and potentially serve as a reliable proxy for thermoregulation, helping to assess the risk of exertional heat illness^2^. However, the literature on influential factors and intra-individual reproducibility on IRT measurements during exercise remains scarce.

Applying IRT in the context of endurance exercise may provide real-time insights into athletes internal load^2,5–9^. Notably, the thermal pattern of perforator vessels appears across the body during endurance exercises and varies locally. Recently, this pattern has been proposed to be of particular interest in field test scenarios^10^. In addition, thermal pattern distribution and entropy analysis in the chest region have been demonstrated to be associated with cardiorespiratory fitness^11^. Interestingly, this pattern was already analysed in 1992 but was not further investigated in subsequent studies^12^.

In running, Belinchón-deMiguel et al.^13^ reported that T_SK_ variations detectable by IRT are related to physiological and biomechanical load during an ultra-endurance race. Similarly, another study found that IRT captures acute metabolic stress responses during racewalking and provides insights into individual physiological adaptation^7^. In rowing, Silva et al.^14^ demonstrated that high-intensity rowing induces distinct thermal responses in the upper and lower body. Increased temperature in active muscle groups reflects elevated metabolic activity and blood flow, whereas cooler skin regions indicate reduced perfusion and muscle recruitment. Overall, thermal pattern distributions are influenced by thermoregulatory mechanisms such as sweating and blood redistribution. In cycling, Kapoor et al.^15^ and Jastrzębska et al.^16^ reported significant correlations between aerobic fitness and T_SK_ variations. Further studies have demonstrated that thermal pattern distribution and entropy analysis of thermal images can be used to assess physiological stress responses during cycling^11,17^. Novotny et al.^18^ showed that specific muscle activity patterns in breaststroke swimming can be visualised using IRT, indicating that IRT can be utilized for biomechanical analysis in swimmers. In addition, IRT was able to detect differences in thermal responses to endurance exercise in cross-country skiers and swimmers, which were associated with sport-specific physiological demands^6^.

The current scientific debate on applying IRT in endurance performance diagnostics focuses on whether it can detect acute physiological responses and on the influence of factors, such as sweat rate^19^, T_CORE_^20^, ambient temperature^21^, region of interest (ROI)^1,22,23^, exercise intensity^3^, circadian rhythm^24^, and subcutaneous fat^25,26^. T_CORE_ in particular is contested, both with respect to the use of IRT as non-invasive indicator of T_CORE_ and regarding the influence of T_CORE_ on T_SK_ variations during exercise^1,20,27,28^. It has been proposed that specific ROIs are not suitable for indicating changes in T_CORE_^1,27^. Furthermore, although no significant correlations between T_CORE_ and T_SK_ variations have been observed^28^, the core-to-skin temperature gradient is a physiologically relevant marker of thermoregulatory status that is affected by aerobic capacity^29^. This gradient is usually assessed using skin-mounted temperature sensors^29–31^, and IRT-based evaluation has so far been limited to the clinical setting of septic shock^32^. However, measurements of T_CORE_ variations under thermoneutral conditions is frequently not included in IRT experiments, highlighting the need for further research in this area.

In summary, the available findings are promising, but the research landscape in the field is characterized by heterogeneous study designs, which reduces the comparability of results and hinders standardization. In addition, investigations into the use of IRT across different endurance exercise protocols and in retest scenarios are limited, thereby impeding the validation of associations between T_SK_ variations and physiological traits, and intra-individual reproducibility^2^^3^.

Recently, advanced deep learning-based automatic analysis of thermograms of the posterior legs captured during running has been demonstrated, enabling objective and reproducible time series analysis of several statistical variables in specific ROIs to comprehensively explore synchronized cardiopulmonary and thermographic data^33–35^. Therefore, IRT coupled with artificial intelligence-driven automatic analysis is required to provide further evidence for preliminary assumptions and to enable objective thermogram analysis. Additionally, the calves appear to be a preferable, practical, and promising ROI in running and cycling IRT experiments^1,36^.

In the context of the current state of research, three exploratory research objectives were defined for this study. The aim was to explore: (i) the association of distinct T_SK_ metric variations with cardiopulmonary responses during different running protocols with a similar average external load, (ii) the potential physiological and environmental factors that influence T_SK_ variations and (iii) the reproducibility of inter- and intra-individual T_SK_ variations under thermoneutral conditions.

## Methods

### Study design and participants

**Fig. 1:**
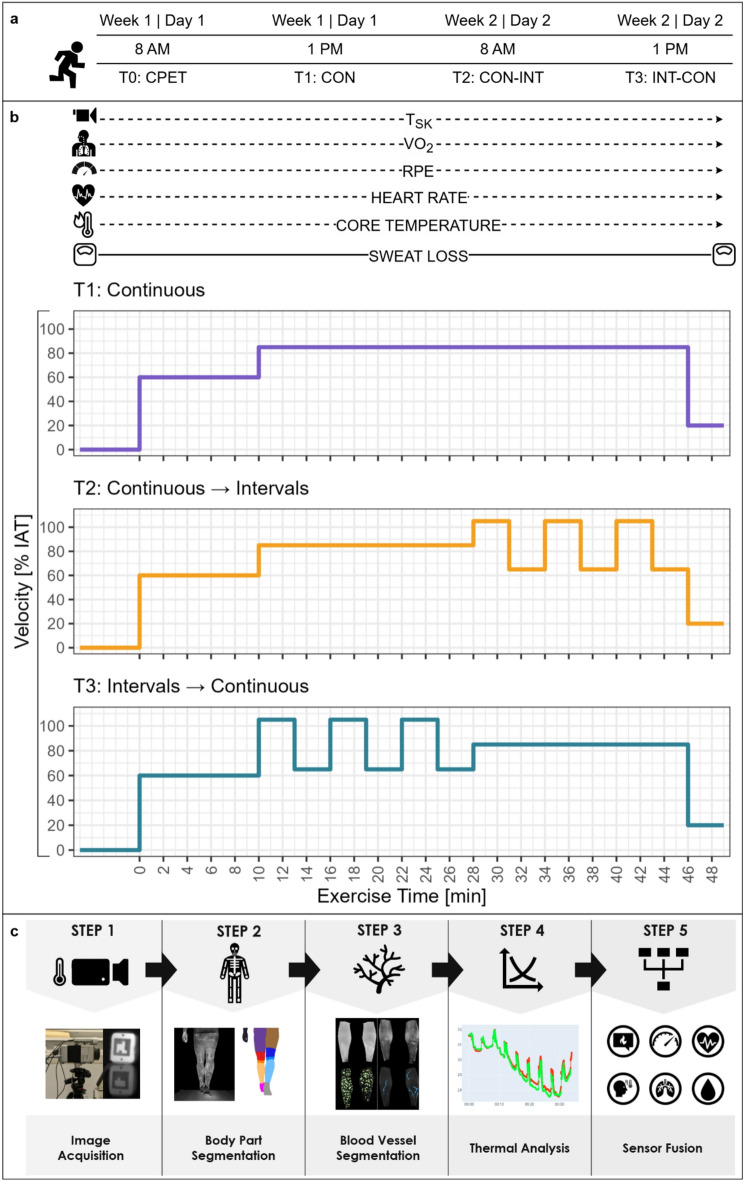
Overview of the study design and data processing pipeline. (**a**) Timeline of the baseline cardiopulmonary exercise test (CPET) and the three exercise sessions (T1, T2 and T3) with continuous (CON) and intermittent (INT) external load. (**b**) Overview of the three running protocols and the measured parameters during the exercise sessions. The different skin temperature (T_SK_) metrics, oxygen consumption (VO_2_), rate of perceived exertion (RPE), heart rate and core temperature were continuously measured throughout the exercise (dashed arrows) and sweat loss was measured before and after the exercise (solid line). (**c**) IRT data processing pipeline from Andrés López et al.^35^.

Participant recruitment and examinations were conducted between March and June 2022. The participants performed three running sessions and one incremental cardiopulmonary exercise test (CPET) on a treadmill in the laboratory of the Department of Sports Medicine, Prevention and Rehabilitation at Johannes Gutenberg-University in Mainz, Germany. In the first week, they performed the initial CPET (T0) to exclude exercise-induced cardiovascular risks and to determine the external load for the subsequent three submaximal running sessions. In the afternoon of the same day, they performed a continuous running session (T1). In the following week, participants performed their third (morning, T2) and fourth (afternoon, T3) running sessions, consisting of continuous and intermittent load within a single day. During all experiments, cardiopulmonary, metabolic, and thermo-physiological parameters were monitored synchronously and continuously (Fig. 1). Healthy individuals aged 18–30 years with a physical activity rating of at least 6 points were eligible for inclusion. Individuals with any of the following characteristics were excluded: inability to perform exercise, acute injury, extensive scars or tattoos on relevant ROIs, body mass < 40 kg, intestinal disease, increased risk of intestinal disease, motility disorders of the gastrointestinal tract, surgical interventions in the gastrointestinal tract, swallowing disorders (e.g., gag reflex), chronic disease, use of acute or chronic medication, pacemaker or electromedical implants, or pregnancy. By informing the participants in advance and using an additional medical history form prior to the examination, the participation of individuals who did not meet the criteria was prevented.

Sixteen endurance-trained individuals who self-reported being healthy at the time of recruitment were enrolled in the experiment. Twelve of the sixteen completed all running sessions. Four individuals were excluded after CPET (T0) due to medical issues (e.g., suspected post covid-19 condition), and one individual was removed prior to data analysis due to irregular non-uniformity temperature drift correction. Consequently, the final analytical sample comprised 11 healthy participants without any diagnosed or identified medical conditions. All procedures performed were reviewed by the Ethics Committee of the State Medical Association of Rhineland-Palatinate and complied with the Declaration of Helsinki (ethical approval identifier: 2021–15713). Additionally, this exploratory single-arm study was registered on 23 June 2022 in the German Clinical Trial Register (clinical trial number: DRKS00029114; URL: https://drks.de/search/en/trial/DRKS00029114). All participants were informed about the study procedures and provided written informed consent.

### Baseline assessment and cardiopulmonary exercise testing

At baseline, participants rated their habitual physical activity (PA-R)^37^, underwent a resting pulmonary function test (Bodybox 5500, MEDISOFT GmbH, Hamburg, Germany), and a bioimpedance analysis (InBody 3.0, Biospace, Seoul, Korea). Afterwards, they performed a stepwise incremental CPET on a treadmill (Saturn, HP cosmos, Nussdorf-Traunstein, Germany). The CPET protocol started after a 10-min acclimatisation period at 6 km/h with an incline of 1.5%. After every 3-min stage, separated by a short resting break (45 s), the velocity was increased by 2 km/h until maximum voluntary exhaustion. During the CPET, respiration was measured by breath-by-breath analysis (Ergostik, Blue Cherry, Geratherm Respiratory GmbH, Bad Kissingen, Germany), heart rate (HR) was recorded with a chest-strap monitor (Polar H10, Polar Electro, Kempele, Finland), and rate of perceived exertion (RPE) was assessed using the BORG scale [6–20]^38^. Capillary blood samples were obtained from the earlobe at rest, after the end of each intensity stage, and at volitional exhaustion. Blood lactate concentration was analysed immediately on site (EKF-diagnostic GmbH, Magdeburg, Germany), and the individual anaerobic threshold (IAT) was determined according to Dickhuth’s model^39^. Adverse events occurring during the examination procedures were monitored and recorded for all participants.

### Three different running protocols with the same average external load

Each running protocol began with a 10-min acclimatisation period in a standing resting position on the treadmill, followed by a 10-min warm-up period with an external load equivalent to 60% of the running velocity at the IAT (vIAT). The incline of the treadmill was set to 1.5% for each running session. After the warm-up, the first running protocol (T1) continued for an additional 36 min at a constant external load (CON) corresponding to 85% of the vIAT and was performed on the first experimental day between 1:00 and 2:30 PM. The second running protocol (T2) consisted of an continuous external load from min 10 to min 28, followed by a intermittent load (INT) from min 28 to min 46. The intermittent part comprised three sequences of 3 min at 105% of vIAT alternating with 3 min at 65% of vIAT. Participants performed this protocol on the second experimental day between 8:00 and 9:30 AM. The third running session (T3) was also conducted on the second experimental day, between 1:00 and 2:30 PM. This protocol included the same 18-min intermittent and continuous running periods, but in reverse order. Each running protocol concluded with a 3-min recovery period of walking at a velocity of 4 km/h. The total duration of each running session was 49 min. In addition to cardiopulmonary parameters, T_CORE_ was measured using ingestible telemetric pills (Telemetric System for Continuous Gastrointestinal Temperature Monitoring, Herouvile ST Claire France) on the second experimental day, and non-invasive T_CORE_ ANT+ sensors (CORE 1 Sensor, greenteg AG, Zürich, Switzerland) on both experimental days. The telemetric pills were ingested 1 h prior to the first exercise session, as Notley et al.^40^ demonstrated no significant differences in core temperature recordings between pills ingested 12, 6, 3–1 h before data collection across rest, exercise and recovery conditions. To avoid confounding temperature measurements, participants were not permitted to consume any food or fluids between the ingestion of the telemetric capsule and the termination of the first exercise session. The individual metabolic heat production rate (MHP [W/m^2^]) was calculated using the metabolic rate obtained through indirect calorimetry, body surface area (BSA [m^2^]), and the external work rate [W]^41^. BSA was estimated using Mosteller’s formula^42^ from the individual body mass and body height. The quantity of sweat lost was calculated as the difference in body mass before and after exercise. Participants were instructed to void their bladders prior to being weighed, in minimal clothing, using calibrated digital scales accurate to 0.1 kg, both immediately before and after the exercise session. No fluids were consumed, and no urine was voided during the exercise session to ensure that the change in body mass accurately reflected sweat loss. An overview of the running protocols, including the measured parameters, is provided in Fig. 1.

### Infrared thermography protocol

The thermal camera specification and calibration routine are described in Andrés López et al.^35^ (VarioCam HD head 800, JENOPTIK AG, Jena, Germany; detector type: uncooled microbolometer with 1024 × 768 IR pixels, long-wave infrared 7.5–14 μm, 30 frames per second (fps) with rolling shutter; thermal resolution 0.02 K, measurement accuracy ± 1.0 K). The camera was placed 235 cm behind the ROI, and the optical axis of the camera is perpendicular to the ROI surface. The temperature range was set to 25–35 °C and the emissivity to 0.98 by the external two-point calibration device, allowing each thermogram to be consistently calibrated with temperature drift correction. Sources of external heat radiation were minimized by keeping windows and doors closed, blinds lowered, and the number of people in the room was kept constant to avoid influencing T_SK_ by drafts and to maintain stable laboratory conditions. Room temperature [°C] and humidity [%] were measured using a hygrometer (Klimalogg Pro Thermo-Hygro-Station, TFA-Germany, Wertheim-Reichholzheim, accuracy ± 1 °C and ± 3% relative humidity). Before starting the measurements, the laboratory was cooled down to temperatures ranging between 19.7 ± 0.97 °C and 20.7 ± 0.89 °C, with humidity levels between 42 ± 5.1% and 45.3 ± 6.3%. This cooling process was undertaken to ensure thermoneutral conditions, with the objective of focusing on exercise-induced thermoregulatory responses while minimizing the impact of confounding environmental thermal stress. This approach is consistent with the majority of studies examining exercise-induced skin temperature changes^2^. Prior to the measurement days, participants followed a strict preparation protocol to standardise external and internal influential factors that could affect the procedures. Strenuous physical activity and the use of skin care and shaving products were prohibited in the 24 h before the experiments. Further instructions included not showering on the morning of testing; abstaining from food and drink for 2 h before the experiment; limiting fluid intake to ≤ 2 L in the preceding 10 h; avoiding caffeinated beverages and alcohol; and obtaining at least 8 h of sleep the night before. Travel to the laboratory should minimize physical exertion (car or public transport). During testing, participants wore standardized clothing: running shoes, ankle socks, knee-length (or shorter) running shorts and, where applicable, a sports bra; long hair was to be secured with a hair tie.

### Deep learning-assisted automatic thermogram analysis

The processing pipeline by Andrés López et al.^43^, from acquisition to deep neural network (DNN) analysis, executed ROI extraction of the whole calf on both legs followed by the identification of blood vessel-related patterns of superficial veins and cutaneous arterial perforator vessels. As a further development of previous work, the semantic segmentation models were optimized using 670 training images and were evaluated using 200 test images for both model types—body parts and blood vessels. We applied a DeepLabv3 + ^44^ model for the body part network, with the following hyperparameters: regional mutual information loss^45^, a learning rate of 0.000686, and a batch size of 8 optimized using the AdamW algorithm^46^. The vessel network was implemented using an Attention-U-Net^47^ architecture, a Tanimoto loss function^48^, a learning rate of 0.000052 and an AdaBelief^49^ optimization algorithm. Finally, four different T_SK_ metrics were calculated: mean surface radiation temperature (T_MEAN_), non-vessel surface radiation temperature (T_NV_), perforator surface radiation temperature (T_P_) and vein surface radiation temperature (T_V_), and their associated Shannon entropy of pixel intensities^50^.

### Integration of IRT and sensors

For comparative analysis, data from various measurement systems, including IRT, HR monitors, ingestible T_CORE_ pills, ANT+ T_CORE_ sensors, metabolic analyzers, and environmental data, were combined into a single dataset. Although the IRT data provides 30 fps, not all thermograms offer the same level of insight. The analysis focused on the stance phase of a single step, when the leg is in extension. The swing (flight) phase was excluded due to substantial motion blur and frequent occlusions of the ROI. Valid images were selected individually by identifying the largest segmented calf in a series of images and treating these images separately. The low-frequency sensor data were then resampled to align with the timing of the high-frequency IRT data. If necessary, a Savitzky–Golay filter with a window length of 151 was applied to smooth the IRT and sensory data and reduce high-frequency noise. To provide an overview of a single experiment, each stage was represented by a single data row corresponding to the mean value over the final five seconds of the stage. The acceleration and deceleration phases of the treadmill were excluded. Data integration was performed using python (v3.8.13), pandas (v2.0.3), OpenCV (v4.8.1), and SciPy (v1.10.1). DNN training was carried out with torch (v2.4.1) and lightning (v2.3.3) on a CUDA-enabled device.

### Statistical analysis

All statistical analyses and visualizations were conducted using R (v4.4.3) and the integrated development environment RStudio (v2025.05.1 + 513 for Windows). Numeric variables are generally presented as mean ± standard deviation (SD), and categorical variables are expressed as n (%). All visualizations were generated using the *ggplot2* package (v3.5.1), except for the heatmaps which were created with the *ComplexHeatmap* package (v2.22.0). Data exclusions were applied due to technical issues or missing data points. The following combinations of participant, running session and phases were excluded from the heart rate specific analyses: P9/T1 (all phases), P1/T2 (second half), P6/T2 (second half), P1/T3 (second half) and P4/T3 (second half). For VO_2_, the first half, second half, and recovery phases of P10/T2 were excluded. Core temperature data from the ANT+ sensor were excluded for P9/T1 (all phases), and ingested pill sensor data were excluded for P4/T2 (all phases). The statistical significance level was set at *p* < 0.05.

To visualize the mean T_SK_ metrics for all participants a 3rd-order digital low-pass Butterworth filter was applied using the *signal* package (v1.8.1) to suppress high-frequency noise, with a cutoff frequency of 0.04 Hz normalized by the Nyquist frequency. First, the relevant variables were aggregated by taking the median across participants at each timepoint. Subsequently, a rolling median filter with a window width of 350 samples was applied to further reduce noise. For a specific variable (T_V_), missing values were first linearly interpolated using the *zoo* package (v1.8.12) prior to filtering. To investigate the associations between temperature and physiological variables across multiple stages, we performed a systematic correlation analysis using parametric and non-parametric approaches, depending on the data distribution. Normality was assessed using the Shapiro–Wilk test with the *stats* package (v4.4.3). Pearson or Spearman rank correlation were then applied using the *stats* package as appropriate. When multiple data points from the same measurement were included in the analysis, the *rmcorr* package (v0.7.0) was used to account for variability in the intercepts across individuals. We report the repeated-measures correlation coefficient (r_rm_), the corresponding 95% confidence intervals (95% CI, via bootstrapping), and the significance levels. Correlation strength was interpreted according to Cohen’s conventions, with *r* < 0.1 trivial, 0.1–0.29 small, 0.3–0.49 moderate and ≥ 0.5 large^51^.

We fitted linear mixed-effects models using the *lmerTest* package (v3.1.3) to examine the effects of experimental conditions on T_SK_ metrics, T_CORE_ and RPE and to identify differences in ΔT_NV_ across the different running sessions. Participant was included as random intercept to account for repeated observations within individuals. Fixed-effects structures followed the a priori design, including the relevant within- and between-subject factors and their necessary interactions. When factors had more than two levels, pairwise comparisons were obtained with the *emmeans* package (v1.11.0) using Tukey’s honest significance difference adjustment for multiple testing. Model assumptions were evaluated using the *performance* (v0.15.2) to generate residual-versus-fitted, Q-Q plots, and related diagnostic plots, and were complemented with simulation-based residuals checks from *DHARMa* (v.0.4.7), including formal tests for outliers and dispersion. If diagnostics indicated violations such as non-normal residuals, heteroscedasticity, or influential outliers, we refitted the model with *robustlmm* (v3.3.1) to obtain robust estimates and confirmed that the overall pattern of fixed-effect results was stable across the two approaches.

We calculated the intra-class correlation (ICC) using the *irr* package (v0.84.1). Specifically, we used a two-way mixed-effects model (model = “twoway”) to estimate ICC based on the chosen ICC type (e.g., “consistency” or “agreement”) and calculated the reliability of single measurements (unit = “single”), which is suitable when individual observations rather than average scores are of interest. ICCs were calculated for each individual during six different exercise sections [T1-T2-T3 (warm-up), T1-T2 (CON), T1-T3 (CON), T2-T3 (CON), T2-T3 (INT), T1-T2-T3 (recovery)]. The interpretation of ICC values followed conventional thresholds: values below 0.5 indicated poor reliability, values between 0.5 and 0.75 indicated moderate reliability, values between 0.75 and 0.9 indicated good reliability, and values above 0.9 indicated excellent reliability^52^.

## Results

All individuals included were of normal weight, with a BMI ranging from 20.5 to 24.3 kg/m^2^, and a body fat percentage ranging from 10.2 to 18.1% for male participants, and from 13.6 to 25.3% for female participants.

The participants were well-trained, characterized by VO_2max_ between 41 and 65.9 mL/min/kg and vIAT between 9.9 and 16.5 km/h, and were engaged in intense exercise on a regular basis, as indicated by their PA-R scores ranging from 6 to 10 points (Table 1). No adverse events were observed during the study procedure.

**Table 1:** Characteristics of the participants.

*n*	Sex	Age [y]	Body height [cm]	Body mass [kg]	BMI [kg/m^2^]	Body fat [%]	Lean mass [kg]	BSA [m^2^]	PA-*R* [1–10]	vIAT [km/h]	VO_2max_ [mL/min/kg]
1	m	25	178	76.3	24.1	15.1	60.8	1.94	10	16.5	65.9
2	f	27	179	68.4	21.3	15.8	53.9	1.84	10	13.3	47.5
3	m	27	178	67.6	21.3	11.6	56	1.83	8	12.2	48.8
4	m	29	181	83.6	24.1	10.2	66.6	2.05	7	12.2	59.2
5	f	22	167	57.1	20.5	20.3	42.5	1.63	9	12.5	50
6	m	22	170	70.4	24.3	18.1	54	1.82	9	13.3	57.9
7	f	23	160	60.5	23.6	21.3	44.5	1.64	10	10.7	45.9
8	m	23	177	72.6	23.1	14.1	58.4	1.89	6	11.2	56.9
9	f	26	166	58.9	21.4	25.3	41.1	1.65	7	9.9	41
10	f	24	175	65.2	21.3	13.6	52.8	1.78	7	10.4	41.8
11	f	24	177	72.2	22.8	18.1	54.7	1.88	9	12.5	49.2
Mean ± SD		24.7 ± 2.3	173.5 ± 6.7	68.4 ± 7.9	22.5 ± 1.4	16.7 ± 4.5	53.2 ± 7.8	1.8 ± 0.1	8.4 ± 1.4	12.2 ± 1.8	51.3 ± 7.8

*BMI = body mass index; BSA = body surface area; PA-R = physical activity rating; vIAT = velocity at individual anaerobic threshold; VO_2max_ = relative maximum oxygen uptake.

### **Response of T**_SK_**to varying external load in comparison to established physiological markers**

**Fig. 2:**
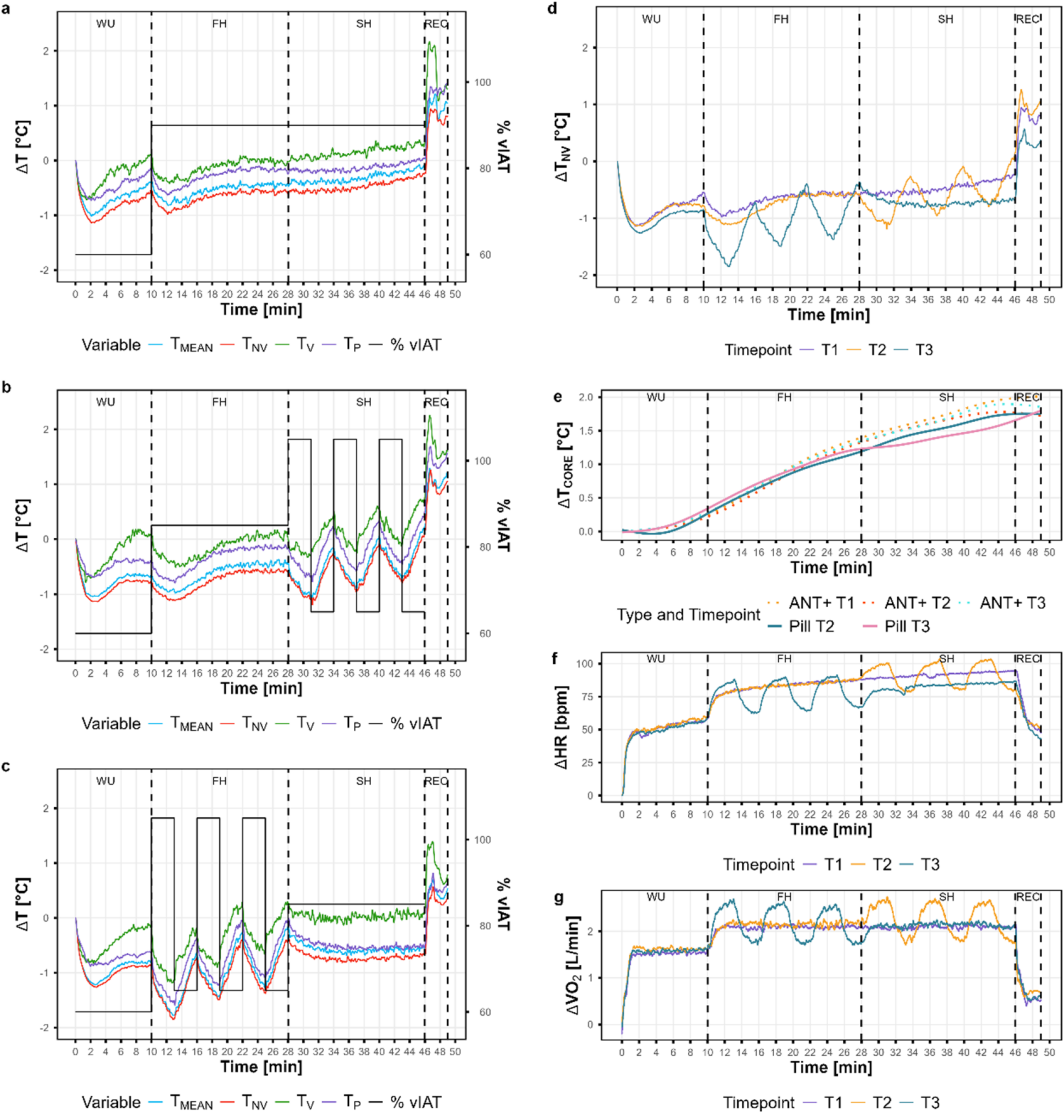
Synchronized time series data for all three running sessions, averaged over the study cohort. X-axis represents exercise time and different load sections: warm-up (WU), first half (FH), second half (SH), recovery (REC) are separated by vertical dashed lines. The mean temperature (T_MEAN_), non-vessel temperature (T_NV_), vein temperature (T_V_) and perforator vessel temperature (T_P_) are presented for the different running sessions consisting of continuous running (T1) (**a**), continuous-intermittent running (T2) (**b**) and intermittent-continuous running (T3) (**c**) with the external load protocol (oriented on the running velocity at the individual anaerobic threshold [%vIAT]) as second Y-axis. The variations of T_NV_ (**d**), core temperature (T_CORE_) measured with an ANT+ sensor and telemetric pill (**e**), heart rate (HR) (**f**) and oxygen consumption (VO_2_) (**g**) are presented in a single panel for all three running sessions. Excluded tests due to measurement errors: T_CORE_-ANT + *n* = 1, T_CORE_-PILL *n* = 1, HR *n* = 5, VO_2_ *n* = 1.

Overall, all T_SK_ metrics followed the variations in external load during the three running sessions, but systematic differences were observed between the metrics (Fig. 2a–c).

The T_SK_ metrics maintained a consistent order (T_V_ > T_P_ > T_MEAN_ > T_NV_) throughout all running sessions. During the warm-up, all T_SK_ metrics decreased immediately within the first 2–3 min, with T_NV_ showing the greatest reduction, ranging from − 1.13 to − 1.27 °C. From approximately 3 min after exercise onset, T_SK_ increased for all metrics, with T_V_ showing the steepest increase and reaching approximately baseline levels by the end of the warm-up period. In contrast, T_NV_ remained − 0.55 to − 0.86 °C below baseline values at the end of the warm-up phase. T_SK_ variations during the continuous phase were comparable between T1 and T2. When the continuous load was applied during the second half of the session, T_NV_ further increased in T1, whereas a decrease was observed in T3, in which the intermittent phase preceded the continuous load. During the intermittent phase, a more pronounced variation in T_NV_ was observed in T3, where participants had only a 10-min warm-up beforehand, compared to T2, where participants had already run for 28 min before the first interval (− 0.45 °C, − 0.08 °C and − 0.26 °C during the 1st, 2nd and 3rd interval, respectively). In line with the different load protocols, during the second half of the running session, T_NV_ showed the greatest decrease during continuous load in T3 (− 0.66 °C). Conversely, T_NV_ slightly increased during intermittent load in T2 (+ 0.13 °C). All T_SK_ metrics increased immediately after exercise termination, with the highest increase after T1, ranging from 1.18 to 1.81 °C for T_NV_ and T_V_, respectively. The mean temperature increases during this period for all four T_SK_ metrics were comparable between T2 and T3 (1.2 ± 0.19 and 1.21 ± 0.06 °C, respectively), whereas T1 showed a greater increase of + 1.43 ± 0.27 °C. When comparing T_NV_ variations with other physiological parameters—T_CORE_, HR and VO_2_—HR shows the strongest inverse association (Fig. 2e–g). The most pronounced reduction in HR during the recovery phase occurred after T1 (− 48 bpm), corresponding to the greatest increase in T_NV_ after T1. In contrast, the smallest decrease in HR (− 29 bpm) was observed following T2. T_CORE_ increased continuously throughout all running sessions to a similar extent in T1, T2, and T3. VO_2_ varied in line with the external load, with a similar magnitude of variation in all three running sessions, regardless of the prior acute load.

No differences between running sessions after each running phase (WU, FH, SH, REC) were found for T_CORE_ and T_NV_; however, T_NV_ data showed tendencies to differ between the tests at the end of the exercise, which are attenuated by large confidence intervals (CI; Supplementary Fig. S5). Only RPE showed a significant interaction effect between exercise session and running phase (*p* < 0.001; η^2^_p_:0.28). Significant differences between running sessions were observed for RPE after the first half of running in T3 compared with T1 (Δ = − 3.1, SE = 0.711, *p* = 0.002) and T2 (Δ = − 3.5, SE = 0.711, *p* < 0.001). At the end of the exercise sessions, RPE was significantly lower at T2 than at T1 (Δ = − 3, SE = 0.711, *p* = 0.004).

Repeated-measurement correlation showed consistently strong negative correlations for all individuals during the intermittent phase between T_P_ and both HR (T2: r_rm_ = − 0.7, 95% CI [− 0.77, − 0.6]; T3: r_rm_ = − 0.63, 95% CI [− 0.71, − 0.53]) and VO_2_ (T2: r_rm_ = − 0.72, 95% CI [− 0.78, − 0.64]; T3: r_rm_ = − 0.63, 95% CI [− 0.7, − 0.52]). Furthermore, during the recovery period there were strong negative correlations in all three running sessions with T_P_: T1 (HR: r_rm_ = − 0.8, 95% CI [− 0.89, − 0.65]; VO_2_: r_rm_ = − 0.9, 95% CI [− 0.94, − 0.84]), T2 (HR: r_rm_ = − 0.61, 95% CI [− 0.78, − 0.34]; VO_2_: r_rm_ = − 0.86, 95% CI [− 0.92, − 0.78]) and T3 (HR: r_rm_ = − 0.8, 95% CI [− 0.9, − 0.63]; VO_2_: r_rm_ = − 0.9, 95% CI [− 0.94, − 0.83]). All correlations were significant at *p* < 0.001. During the warm-up, there were consistently strong positive correlations between T_CORE_ and the entropy of T_P_ for all three running sessions, measured with both pill (T2: r_rm_ = 0.56, 95% CI [0.38, 0.7]; T3: r_rm_ = 0.68, 95% CI [0.54, 0.78]) and external ANT+ sensor (T1: r_rm_ = 0.72, 95% CI [0.59, 0.81]; T2: r_rm_ = 0.59, 95% CI [0.43, 0.72]; T3: *r* = 0.83, 95% CI [0.75, 0.89]). All correlations were significant at *p* < 0.001.

Individual differences were observed when comparing T_CORE_ with T_NV_ during the warm-up. The two participants with the highest vIAT exhibited consistent negative correlations regardless of the T_CORE_ measurement method. For these participants, correlation coefficients between T_NV_ and T_CORE_ pill ranged from *r* = − 0.75 to − 0.98 across T2 and T3 (all *p* < 0.05), while for T_CORE_-ANT+, two out of the three running sessions showed statistically significant correlations, with r ranging from − 0.7 to − 0.87 (all *p* < 0.05). The participant with the highest vIAT (16.5 km/h) also showed strong negative correlations during the first continuous running section in T1 (*r* = − 0.71 to − 0.79, *p* < 0.001) and T2 (*r* = − 0.89 to − 0.91, *p* < 0.001) measured with both pill and ANT+. In contrast, strong positive correlations between T_CORE_-ANT + and T_NV_ during the warm-up were found for 6 participants at T1 (*r* = 0.83–0.98, *p* < 0.01), 7 participants at T2 (*r* = 0.72–0.98, *p* < 0.05), and at T3 (*r* = 0.81–0.98, *p* < 0.01). During the first continuous running phase, significant strong positive correlations were observed in 6 participants at T1 (*r* = 0.72–0.99, *p* < 0.01) and 9 participants at T2 (*r* = 0.71–0.99, *p* < 0.01), including one of the two individuals who showed negative correlations during the warm-up.

### Environmental and physiological factors affecting T_SK_ variability

**Fig. 3:**
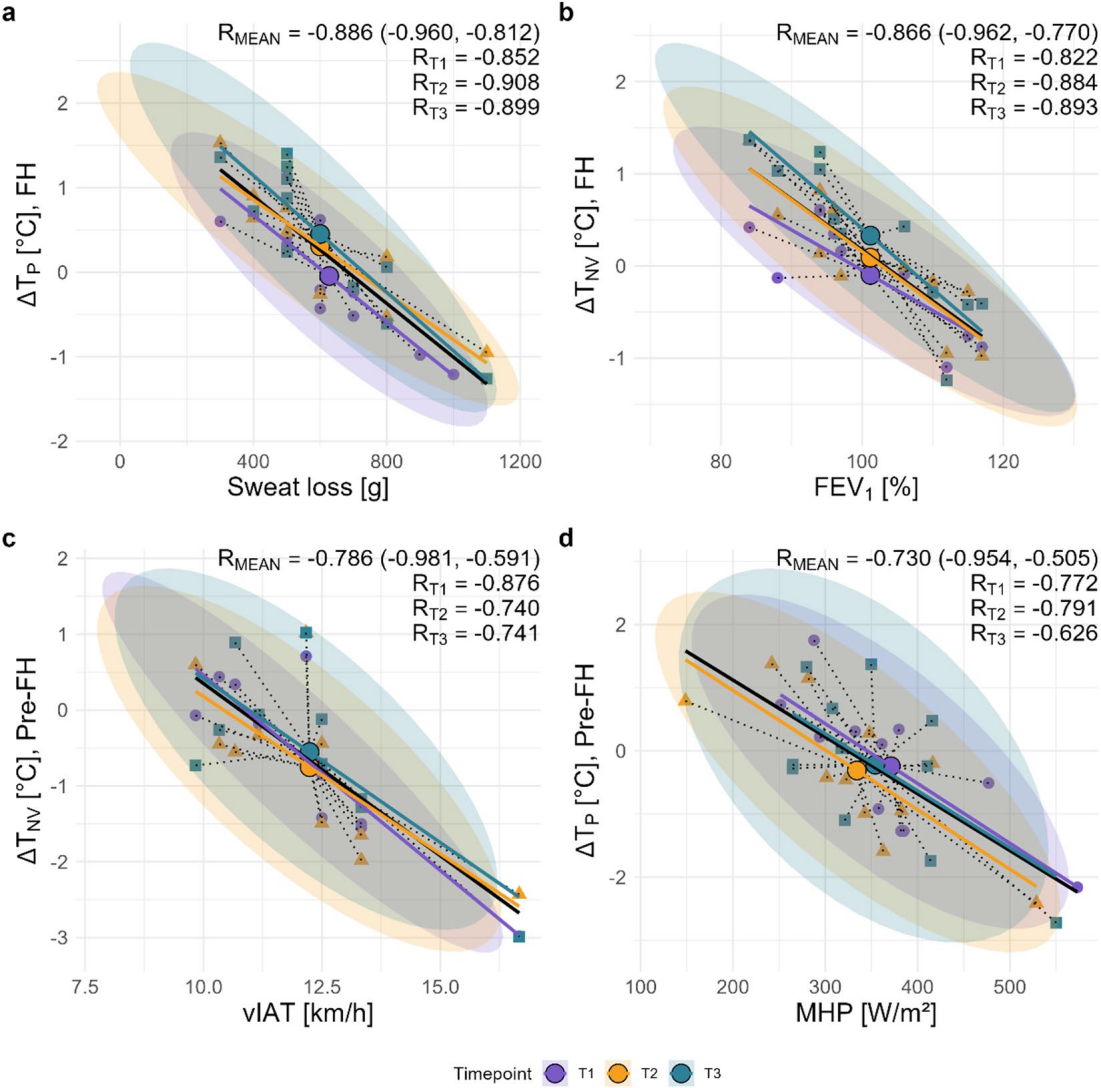
Associations between differences in non-vessel (∆T_NV_) and perforator vessel (∆T_P_) temperature with potential physiological influencing parameters within certain sections of the running protocols. (**a**) ∆T_P_ during the first exercise half (FH) vs. sweat loss [g]. (**b**) ∆T_NV_ during the first exercise half (FH) vs. forced expiratory flow during the first second (FEV_1_) [%]. (**c**) ∆T_NV_ from baseline to the end of the first exercise half (Pre-FH) vs. running velocity at the individual anaerobic threshold (vIAT) [km/h]. (**d**) ∆T_P_ from baseline to the end of the first exercise half (Pre-FH) vs. metabolic heat production (MHP) [W/m^2^] at end of the running session. Data points were color-coded by timepoint (T1, T2, T3), and 95% confidence ellipses were drawn to indicate the distribution of observations within each timepoint. Centroids for each timepoint were calculated and displayed, and the mean correlation coefficients (R_MEAN_) with 95% confidence intervals and correlation coefficients for each individual timepoint (R_T1_, R_T2_, R_T3_) were annotated in the top right corner of each plot.

Further exploratory analyses revealed several significant correlations between T_SK_ variations and physiological factors. Figure 3 presents the four most consistent correlations across the running sessions.

The strongest correlation was found between ΔT_P_ and sweat loss during the FH of the running sessions, with very high consistency across running sessions (*r* = − 0.85 to − 0.91, *p* ≤ 0.001). In the same running phase, a strong negative correlation between ΔT_NV_ and the FEV_1_ was detected across all three running sessions (*r* = − 0.82 to − 0.89, *p* ≤ 0.002). ΔT_NV_ during the Pre-FH phase decreased more with higher vIAT in all three running sessions (*r* = − 0.74 to − 0.88, *p* ≤ 0.009). A strong negative correlation was also found between the ΔT_P_ in the Pre-FH phase and MHP at exercise termination (*r* = − 0.63 to − 0.79, *p* ≤ 0.038). There were strong positive correlations between T_CORE_-pill at exercise termination and both ΔT_P_ from FH to recovery (*r* = 0.70–0.78, *p* ≤ 0.017) and ΔT_P_ during recovery (*r* = 0.62–0.64, *p* ≤ 0.045). T_CORE_-ANT + at exercise termination showed similar correlations with ΔT_P_ from warm-up to recovery (ΔTP WU–REC) at T3 (*r* = 0.85, *p* = 0.002) and a near-significant correlation at T2 (*r* = 0.56, *p* = 0.07), but no association at T1 (*r* = 0.04, *p* = 0.906). VO_2max_ showed a strong negative correlation with ΔT_MEAN_ during the warm-up at T1 and T2 (*r* = − 0.61 to − 0.76, *p* = 0.047–0.014), whereas the correlation at T3 was not significant (*r* = − 0.51, *p* = 0.107). Additionally, VO_2max_ correlated significantly with ΔT_P_ during the first half of exercise at T1 (*r* = − 0.71, *p* = 0.014) and T3 (*r* = − 0.71, *p* = 0.014), but not at T2 (*r* = − 0.45, *p* = 0.169). Environmental temperature was positively correlated with ΔT_P_ Pre-REC, reaching statistical significance only at T1 (*r* = 0.74, *p* = 0.009; T2-T3: *r* = 0.35–0.58, *p* = 0.064–0.317). In contrast, environmental humidity showed no consistent association in any exercise phase.

**Fig. 4:**
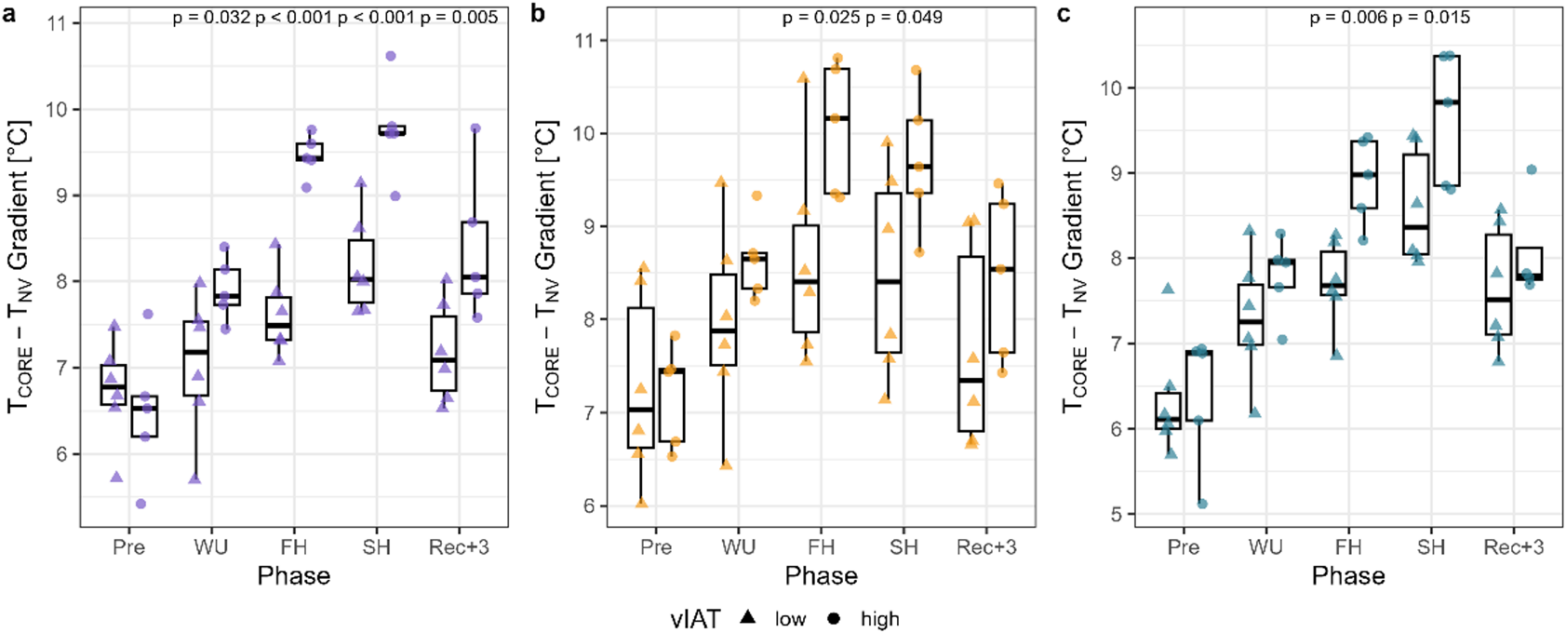
Gradients of core temperature (T_CORE_) and non-vessel temperature (T_NV_) at baseline (Pre), after the warm-up (WU), the first exercise phase (FH), the second exercise phase (SH) and the 3 min recovery phase (REC + 3) between individuals with high and low running speed at the individual anaerobic threshold (vIAT, median split) in running session T1 (**a**), T2 (**b**) and T3 (**c**). T_CORE_ was measured with the ANT+ sensor. Statistical significance: *p* < 0.05 (linear mixed effects model with Tukey’s post hoc test).

Comparing participants with high versus low vIAT revealed no significant differences in T_CORE_ and HR, but significantly higher T_CORE_-T_NV_ gradients at the end of the first half and at exercise termination were observed in all running sessions, measured with an ANT+ sensor (*p* < 0.05, Fig. 4). In contrast, the T_CORE_-T_NV_ gradient did not differ significantly between individuals with high and low VO_2max_ during exercise.

### T_SK_ reproducibility in repeated running sessions

**Fig. 5:**
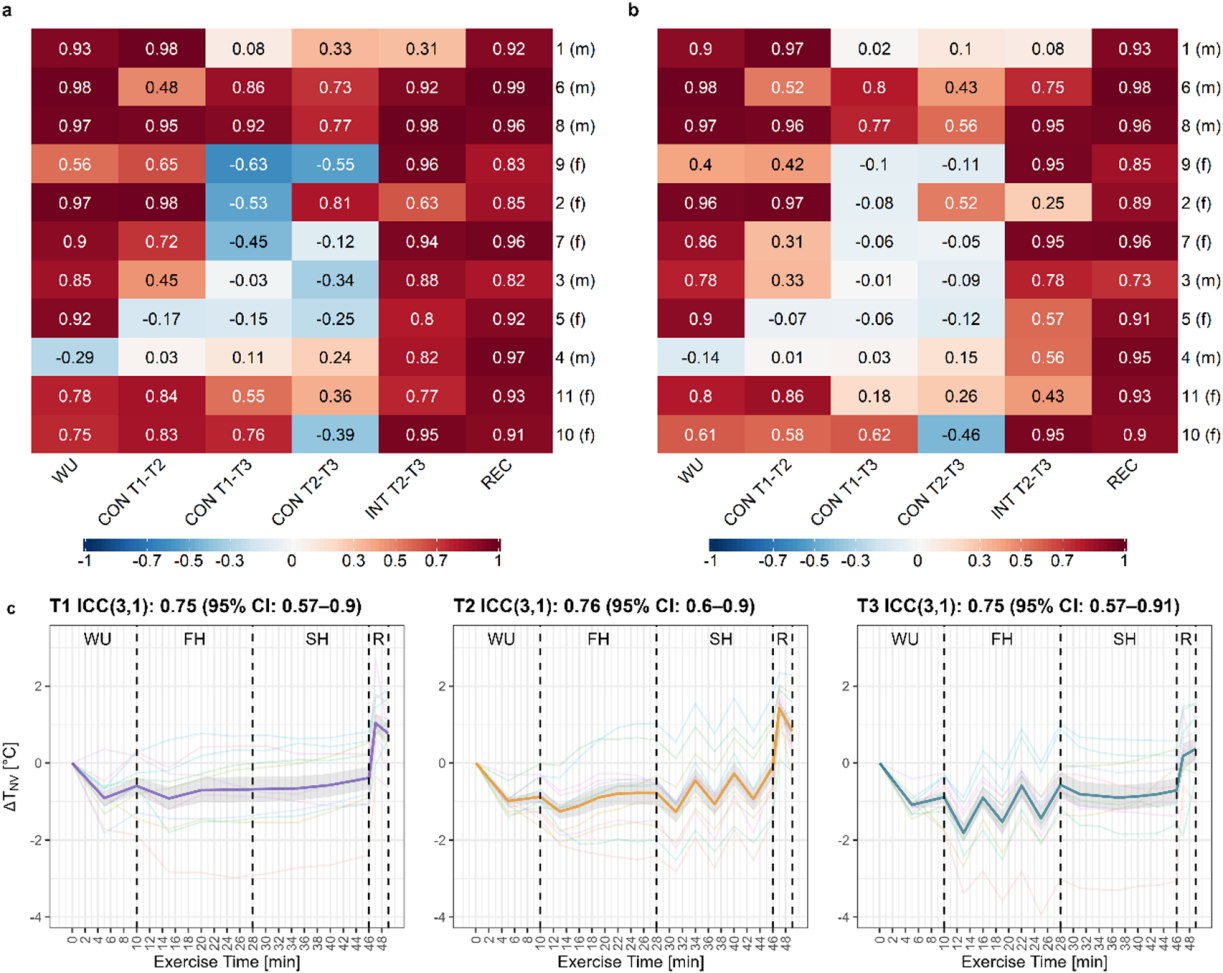
Intra- and inter-individual intra-class correlation (ICC) analysis of the non-vessel temperature (T_NV_). Intra-individual ICC(3,1) for consistency (**a**) and ICC(2,1) for agreement (**b**) during the warm-up (WU), continuous running (CON), intermittent running (INT) and the recovery phase (REC) for each individual as separate row. (**c**) Inter-individual correlation analysis with mean time series and 95% confidence intervals (shaded area) for T1, T2 and T3. *n* = 3 participants are missing T_NV_ data during the recovery phase in T3.

Overall, good to excellent reproducibility was found for the recovery section [ICC(3,1): 0.91 (95% CI 0.9–0.93)], with 8/11 participants showing excellent ICC coefficients (≥ 0.9); for the intermittent sections T2-T3 [ICC(3,1): 0.81 (95% CI 0.79–0.84)], with 5/11 participants showing excellent ICC coefficients; and for the warm-up section [ICC(3,1): 0.76 (95% CI 0.69–0.83)], with 6/11 participants showing excellent ICC coefficients. Moderate ICCs were found for the continuous section T1-T2 [ICC(3,1): 0.61 (95% CI 0.57–0.66)]. Lower ICCs were observed for the continuous sections T1-T3 [ICC(3,1): 0.14 (95% CI 0.07–0.21)] and T2-T3 [ICC(3,1): 0.14 (95% CI 0.07–0.21)], because T_NV_ values during T3 decreased in the continuous section but increased in the corresponding comparison sections. In general, ICC coefficients were higher when analyzing consistency [ICC(3,1): 0.56 (95% CI 0.44–0.68)] compared with agreement [ICC(2,1): 0.52 (95% CI 0.42–0.62)] across all running sections (Fig. 5a and b). Individual time series with ICC(3,1) and ICC(2,1) during every running section can be found as Supplementary Figs. S1 and S2. The inter-individual comparison showed good consistency, with ICC(3,1) ≥ 0.75 for all three running sessions (Fig. 5c).

## Discussion

To the best of our knowledge, this is the first study to continuously examine different variables of skin surface radiation temperature, automatically detected using deep learning-based analysis of thermograms during intermittent and continuous running in endurance-trained individuals. We compared synchronized time series data of four T_SK_ metrics (T_MEAN_, T_NV_, T_V_, T_P_), T_CORE_, RPE, and cardiorespiratory response (HR, VO_2_) during three repeated running sessions with a similar average external load but differing in acute load variation. Based on our analysis, all T_SK_-derived metrics varied in line with changes in external load. The entropy and mean of T_P_ showed stronger correlations with HR, VO_2_ and T_CORE_ than other T_SK_ metrics. Furthermore, T_SK_ measurements during exercise were reproducible at similar external load regardless of day, time of day, or prior exercise session under standardized environmental conditions, and inter-individual differences in T_SK_ variations were associated with submaximal exercise capacity.

When analyzing T_SK_ in relation to external load, the relative variation of all T_SK_ metrics responded immediately and inversely to changes in external load during all three tests, with consistent but distinct absolute values between the T_SK_ metrics (T_V_ > T_P_ > T_MEAN_ > T_NV_). The temperature decrease after exercise onset and the subsequent increase after exercise termination observed across all T_SK_ metrics are consistent with mean temperature responses reported in previous studies^8,53^. Additionally, the spatial differentiation aligns with findings by Arfaoui et al.^54^, who showed that perforator vessels in the gastrocnemius muscle area exhibited higher temperatures than the surrounding ROI during graded cycling exercise. Although all T_SK_ metrics appeared to follow a similar global trend, the analysis revealed differences in their correlation with cardiopulmonary variables. The entropy and mean of T_P_ showed a strong correlation to cardiorespiratory parameters (e.g. HR, VO_2_) during different phases of the running sessions. Similarly, Bogomilsky et al.^11^ and Hu et al.^17^, reported an increase in entropy of the chest surface area that was significantly correlated with exercise duration and intensity during incremental cycling. Furthermore, Masur et al.^55^, reported stronger associations between the kinetics of the thermal contrast index, related to activated perforasomes and internal load parameters than with mean T_SK_ during and following incremental cycling. The high-frequency IRT measurement in the present study revealed an inverse correlation between the entropy of T_P_ and HR and VO_2_ during the intermittent running. This finding suggests short-term alterations in perforator vessels tone between short-term sympathetically mediated vasoconstriction during increases in external load and local vasodilation when external load is reduced, suggesting increased peripheral heat dissipation. Together these findings emphasize the potential for further investigation into different perforasome-associated metrics in the field of endurance exercise, particularly when combined with high-frequency IRT measurements for evaluating short-term physiological alterations during exercise. In contrast to these rapid, load-sensitive T_SK_ variations, T_CORE_ increased continuously throughout the running sessions and reflects cumulative rather than acute thermal strain. Consequently, concurrent monitoring of T_SK_ and T_CORE_ enables differentiation between acute peripheral heat dissipation mechanisms and systemic heat accumulation—a distinction that is, however, highly dependent on environmental context.

The thermoneutral conditions of our experiments ensured that ambient temperature did not confound the T_SK_ metrics, providing a controlled baseline for evaluating the exercise-induced thermoregulatory response. Recently, Martínez-Noguera et al.^56^ observed region-dependent T_SK_ dynamics in racewalkers, with more pronounced T_SK_ decrease at 17 °C compared to stable or attenuated T_SK_ responses at 28 °C, indicating that pre-existing cutaneous vasodilation under heat stress alters the response of IRT-derived metrics during exercise. Likewise, Vainer^57^ reported structurally comparable perforasome patterns after running and sauna exposure, indicating that both metabolically and externally induced thermal stress activate overlapping superficial vascular networks detectable by IRT. Hence, the thermoneutral conditions of the present study likely facilitated more pronounced and less confounded exercise induced T_SK_ responses compared to what would be expected under environmental heat stress, where pre-existing cutaneous vasodilation may attenuate the exercise-induced thermoregulatory response. Notably, exercise modality introduces an additional layer of specificity: Hillen et al.^3^ observed that strength training produces superficial vein prominence, whereas endurance exercise and sauna exposure activate perforator vessel patterns—distinct vascular signatures that our deep learning approach is well positioned to differentiate. Together, these findings suggest that the appropriate T_SK_ metric and its interpretations should be selected with awareness of both exercise mode and environmental conditions, and that the parameters developed here provide a flexible, non-invasive framework for capturing these modality- and context-specific thermoregulatory responses. In the context of these environmental conditions, considerable inter-individual differences in T_SK_ responses were identified that warrant closer examination.

During the warm-up, we observed a positive correlation between the entropy of T_P_ and T_CORE_ across all three running sessions. Interestingly, individual differences emerged when correlating T_NV_ with T_CORE_ during the warm-up and the first continuous running section. Individuals with higher vIAT showed inverse correlations between T_NV_ and T_CORE_, whereas strong positive correlation were found for most individuals with lower submaximal exercise capacity. This is consistent with the much higher standard deviations of T_NV_ during the first primary running phase compared with the second, suggesting different thermoregulatory mechanisms during the early phase of exercise (within the first 28 min), possibly due to a more efficient vasomotor response. In contrast, T_CORE_ exhibited the least inter-individual variability, reflecting a more homeostatic regulation through individual peripheral thermoregulatory mechanisms. To quantify which physiological or environmental characteristics most strongly influence these inter-individual differences, we examined a broader range of potential determinants of T_SK_ variation. Among these, sweat loss, FEV_1_, vIAT and MHP showed the highest negative correlation coefficients, and except FEV_1_, all are closely related to the body’s response to aerobic effort. While both vIAT and VO_2max_ reflect individual endurance performance, vIAT showed a stronger association with T_NV_ (*r* = − 0.749, 95% CI [− 0.55, − 0.87]) than VO_2max_ (*r* = − 0.503, 95% CI [− 0.19, − 0.72]), consistent with the grouped differences observed above. This is in line with evidence that it is not cardiorespiratory fitness (e.g. VO_2__max_) per se that is associated with better heat dissipation, but rather exercise training itself, or more specifically, the repeated thermal stress experienced during training sessions^58^. Accordingly, Samoljanic et al.^59^ reported that running economy, independent of aerobic fitness, alters thermoregulatory responses, with individuals with low running economy showing higher increases in T_CORE_. Furthermore, vIAT is inherently more running-specific than VO_2__max_—individuals with higher vIAT may spend more time training by running than by cycling or swimming, as these latter activities facilitate greater heat loss through conduction and convection, potentially further enhancing their heat dissipation capacity during running specifically. In previous studies, no differences in ΔT_CORE_ were observed between trained and untrained individuals when exercising at a fixed metabolic heat production rate^60^ but skin blood flow has been shown to be elevated in trained compared with untrained at the same relative external load^61^. The finding of higher T_CORE_ - T_NV_ gradients in individuals with higher submaximal exercise capacity across all three running sessions in the present study highlights the importance of T_SK_ measurements for understanding variability in peripheral short-term mechanisms that ultimately lead to similar increases in T_CORE_. This finding is in line with Périard et al.^30^ who reported higher core-to-skin gradients in trained versus untrained individuals. Narrowing these gradients has been shown to increase cardiovascular strain (e.g. increased HR) during moderate intensity cycling^31^. In our cohort, an increased HR response was also observed in the group with lower vIAT, although this did not reach statistical significance. Thus, individuals with higher submaximal exercise capacity appear to exhibit better-developed peripheral thermoregulatory mechanisms during submaximal running exercise than individuals with lower submaximal exercise capacity and therefore may seem to experience reduced cardiovascular strain. While these inter-individual differences are physiologically meaningful, the practical value of continuous T_SK_ monitoring as a tool ultimately depends on its reproducibility across sessions and individuals.

The relative T_NV_ variations during running were reproducible both intra- and inter-individual, regardless of daytime or immediate prior load on the same day, for running sections of identical (warm-up, recovery) or substantial variations in external load (intermittent). However, the reproducibility was weak when comparing continuous load sections of the primary exercise phase. Although the external workload was comparable across conditions, the load was increased immediately prior to the continuous phase in T3, which likely contributed to a reduction in T_NV_ due to acute short-term vasoconstriction. In contrast, no preceding load increase occurred in T1, and in T2 the continuous phase was positioned earlier within the primary exercise segment. These differences in sequencing and prior loading complicate direct comparisons across conditions. In the majority of previous studies, the reproducibility of IRT measurements has been reported only for resting conditions, with ICCs ranging between 0.4 and 0.9^62,63^, without specifying a particular ICC model. One of the highest values (ICC = 0.99) was obtained using computer-aided analysis of thermograms^64^, highlighting the value of automation. During running exercise under hot environmental conditions, James et al.^65^ reported lower ICC values of 0.56 for T_SK_ measurements with IRT in comparison to a telemetry thermistor system (ICC = 0.84) and hard-wired thermistor system (ICC = 0.62). However, that study used a thermal camera model with a substantially lower resolution (160 × 120 pixels), and all measurements were conducted with a hand-held device, factors that may also have contributed to the lower reproducibility compared with the present study. As shown by Machado et al.^66^, the reproducibility of T_SK_ measurements can vary between different thermal imaging camera types. Collectively, these points highlight the necessity for further standardization in the field of thermal imaging in exercise physiology, including IR camera specifications, thermogram calibration, measurement technique, and analysis strategies.

Several influential factors affected the reproducibility of the T_SK_ measurements. From an inter-individual perspective, cohorts with different physiological profiles (e.g., endurance vs. sprint athletes) have been shown to have different T_SK_ variations during running exercise^53^. Therefore, higher inter-individual ICC coefficients might be achieved by investigating a more physiological homogeneous sample (e.g., reduced variability in VO_2__peak_ or vIAT). Interestingly, running phases characterized by greater variability in T_NV_ (warm-up, intermittent, recovery) showed higher ICC than running phases with less variability in T_NV_ (i.e., continuous running during the primary exercise phase). This decline in ICC in low-variability segments is a well-known statistical phenomenon: when a variable lacks sufficient variation, the ICC becomes more strongly influenced by random measurement noise^67^. Additionally, three different time series were included for each participant in the analysis of the warm-up and recovery phases, compared with two time series during the continuous running phases, making the ICC estimates for continuous running more sensitive to random error due to fewer data points.

Future studies should use high-frequency T_SK_ measurements, as static end-point values fail to capture individual short-term thermal responses. This is also supported by the systematic review of Rojas-Valverde et al.^2^, which documented the presence of heterogeneous inter-individual T_SK_ variations. In addition to comparisons of group means, the robust findings on individual differences support individual-specific analyses. Research in thermal imaging requires further standardisation. Addressing methodological discrepancies will enhance study reliability and comparability and advances research in exercise thermoregulation and performance monitoring. Future research could implement reinforcement or unsupervised learning techniques in the DNN-driven processing pipeline to further improve automated vessel detection and the differentiation of different T_SK_ metrics. To generalise our results, the application of DNN-assisted IRT should be replicated in different contexts, including healthy and pathological individuals (e.g., vascular diseases). The subsequent progression in AI-driven analysis will facilitate the automated identification of diseases through artificial classification, while ensuring the preservation of explainability for users.

Despite the strengths of this study, several limitations must be considered when interpreting the findings. The main findings may be generalizable only to young, healthy, and endurance-trained individuals and should be replicated in larger samples with more diverse characteristics. This study was conducted in thermoneutral conditions to focus on exercise-induced thermoregulatory responses. While this approach is consistent with most studies examining skin temperature during exercise, it limits the generalizability of our findings to exercise performed under heat stress or in the cold. Automatic ROI detection during high-velocity running would benefit from improved IR detector capabilities, such as shorter integration times or different shutter types. These improvements would reduce motion blur, missing data points, and noise, leading to enhanced pattern recognition. The image processing pipeline automatically provides objective, reproducible data on predefined features and parameters extracted from thermal images. Nevertheless, investigators must still verify the data output, which requires expert knowledge.

## Conclusion

For the first time, deep learning-assisted IRT provided valuable insights into the interplay and intercorrelation between synchronized skin temperature, core body temperature, perceived exertion, and cardiopulmonary responses during running exercise. The automated thermogram analysis showed consistent intra- and inter-individual T_SK_ variations during repeated running sessions. The robust associations between skin temperature and thermal entropy of the perforator vessels, and body core temperature and sweat loss, highlights the potential for further investigation of skin temperature and its derived metrics. Finally, inter-individual variability in thermoregulatory responses to running exercise appears to be more closely related to submaximal, running-specific performance capacity than to maximal aerobic capacity.

## Supplementary Information

Below is the link to the electronic supplementary material.

Supplementary Material 1

## Data Availability

The data presented in this study are not publicly available but are available on reasonable request from the corresponding author.
