# Supplementary material for: Deep learning-based infrared thermography reveals reproducible uniform and individual thermoregulatory responses during running
Source: Sci Rep. 2026 Mar 28;16:10525. doi: 10.1038/s41598-026-44102-6 (PMC13036059; doi:10.1038/s41598-026-44102-6)
Supplement: Supplementary file 1 — Supplementary Material 1 [file 41598_2026_44102_MOESM1_ESM.docx]

Supplementary Material

Deep learning-based infrared thermography reveals reproducible uniform and individual thermoregulatory responses during running.

Vincent Weber^1^, Daniel Andrés López^2^, David Tobias Ochmann^1^, Severin Zentgraf^1^, Markus Nägele^3^, Elmo Neuberger^1^, Elmar Schömer^2^, Perikles Simon^1^, Barlo Hillen^1,4*^

^1^Institute of Sports Science, Department of Sports Medicine, Disease Prevention and Rehabilitation, Johannes Gutenberg University, Mainz, Germany

^2^Institute of Computer Science. Research Group Computational Geometry, Johannes Gutenberg University, Mainz, Germany

^3^Optoprecision GmbH Bremen, Bremen, Germany

^4^Institute of Occupational, Social, and Environmental Medicine, University Medical Center, Johannes Gutenberg University Mainz, Germany

**Supplemental Figure S1.** Individual time series of all four T_SK_ metrics at T1, T2 and T3 (from left to right).


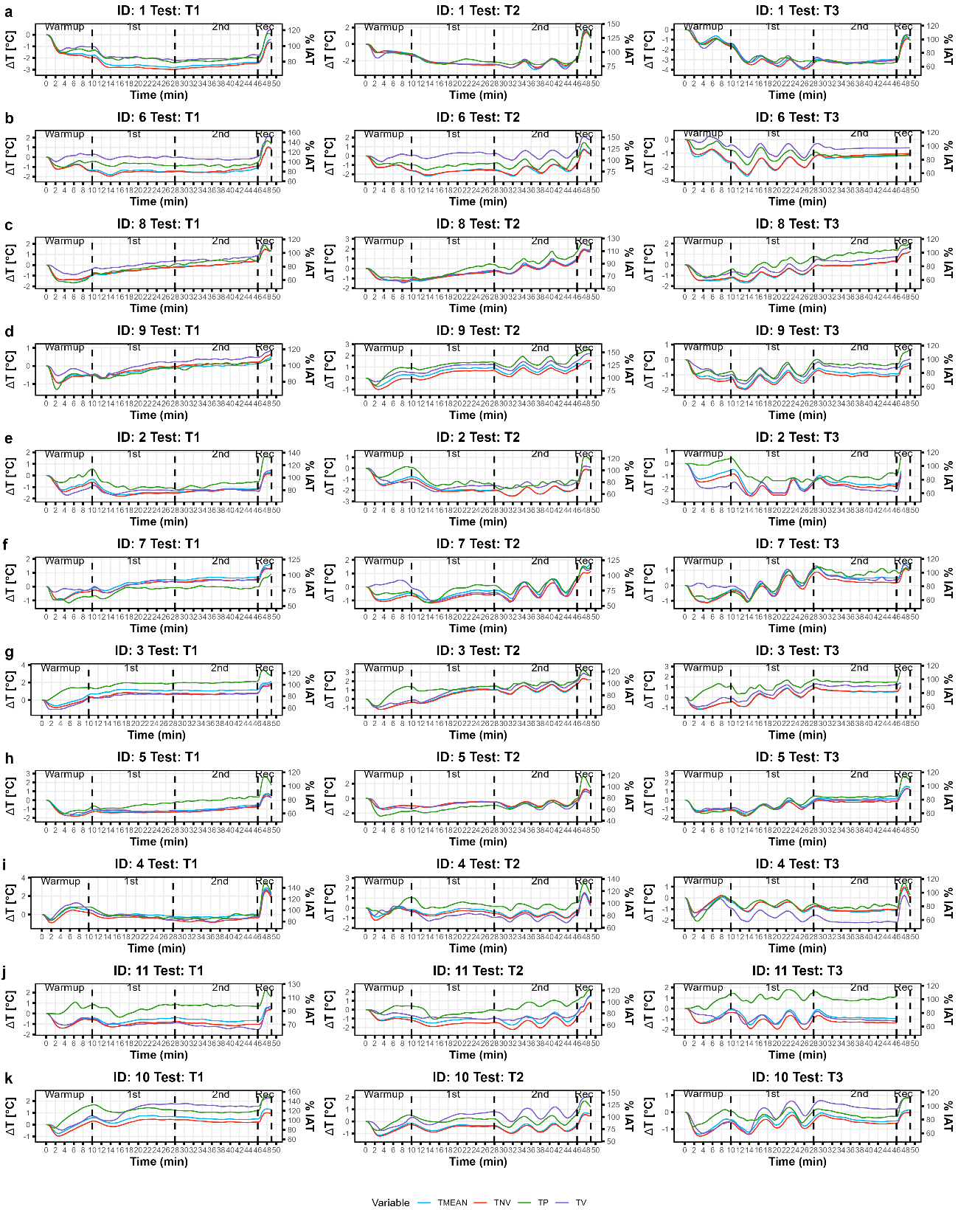


**Supplemental Figure S2.** Individual time series of the non-vessel skin radiation temperature during different running sections with ICC[3,1] for consistency.


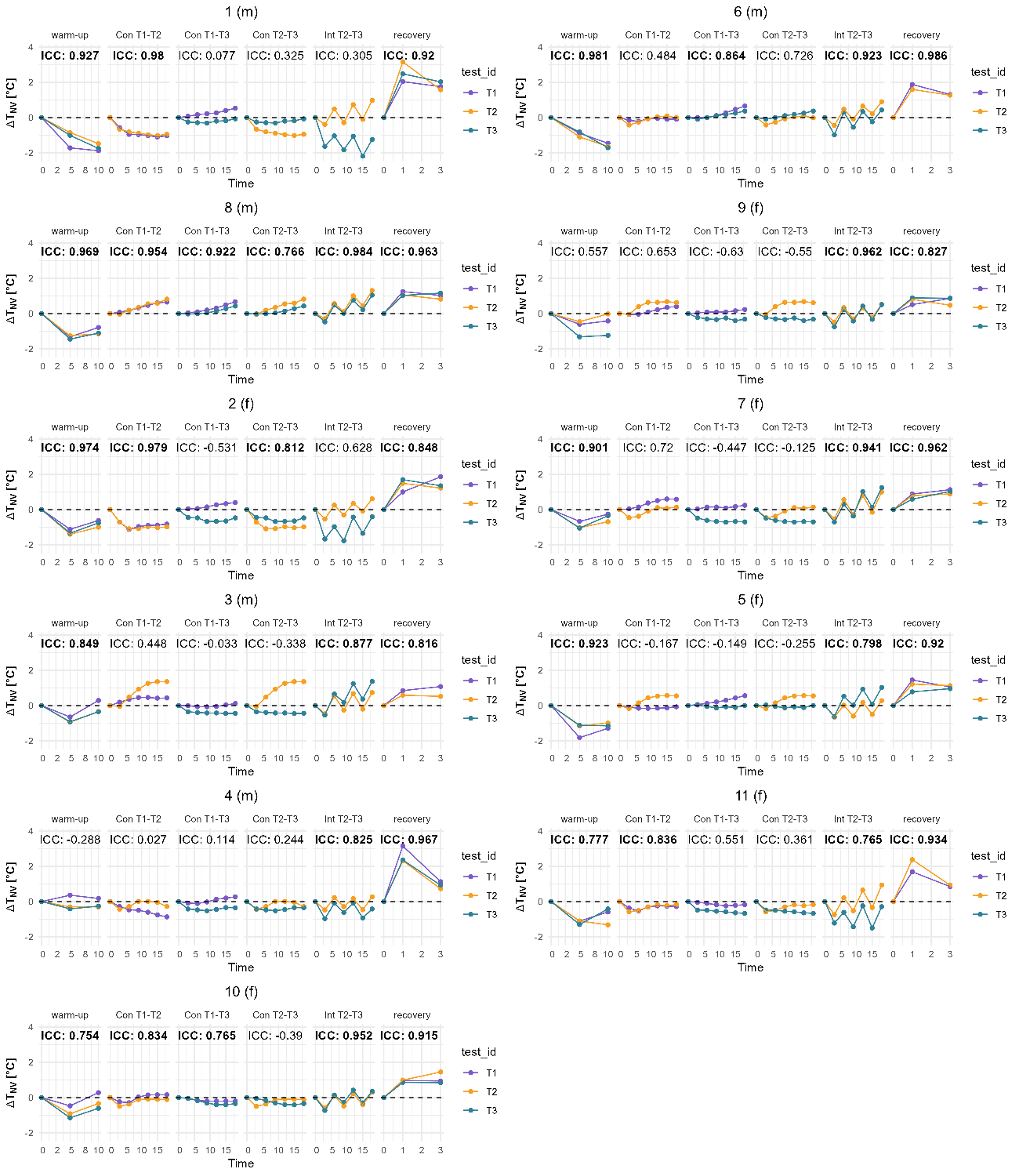


**Supplemental Figure S3.** Individual time series of the non-vessel skin radiation temperature during different running sections with ICC[2,1] for agreement.


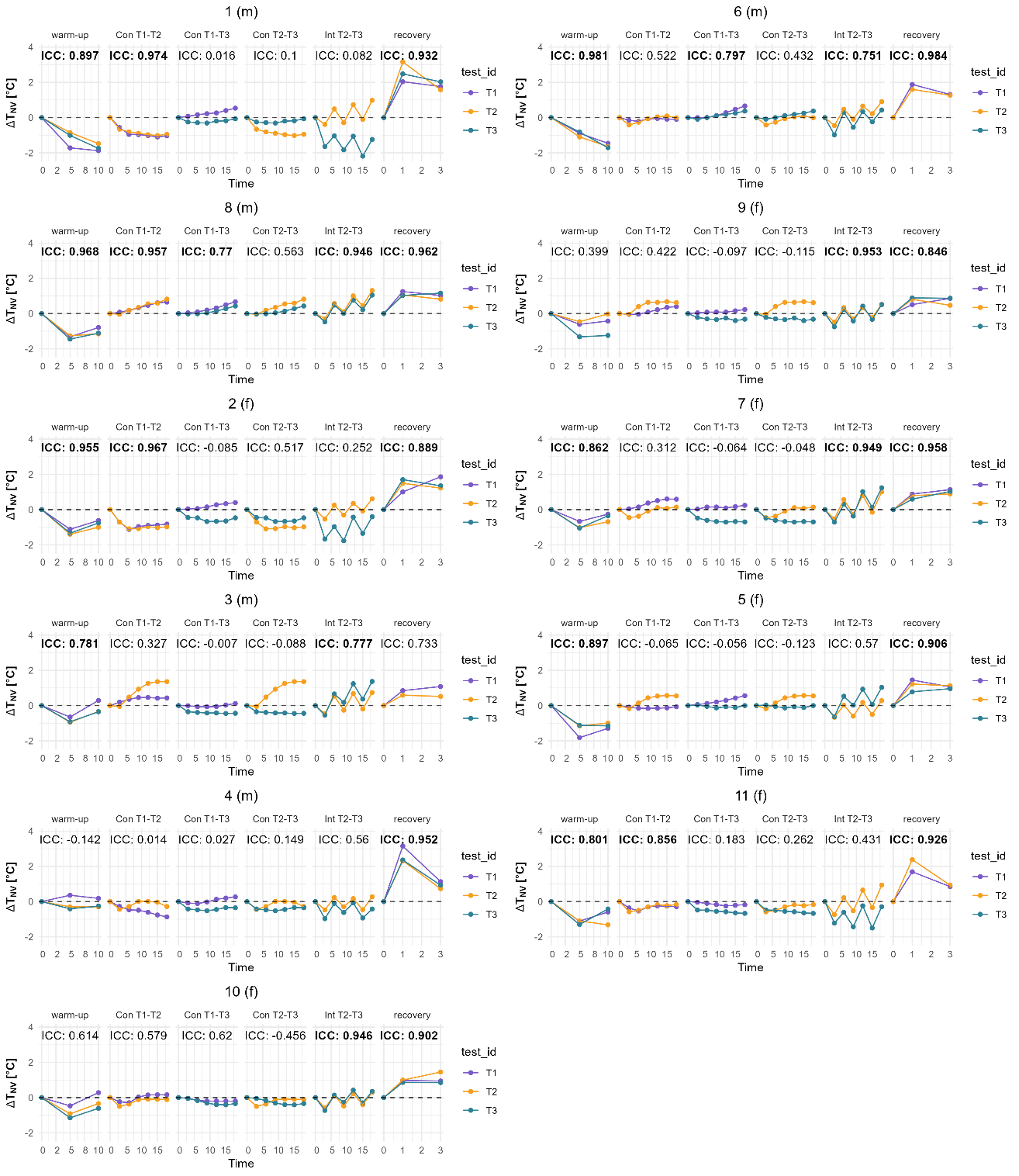


**Supplemental Figure S4.** Automated ROI selection of the individual thermograms for T1, T2 and T3 (from left to right) with the corresponding segmentation mask for T3 on the left.


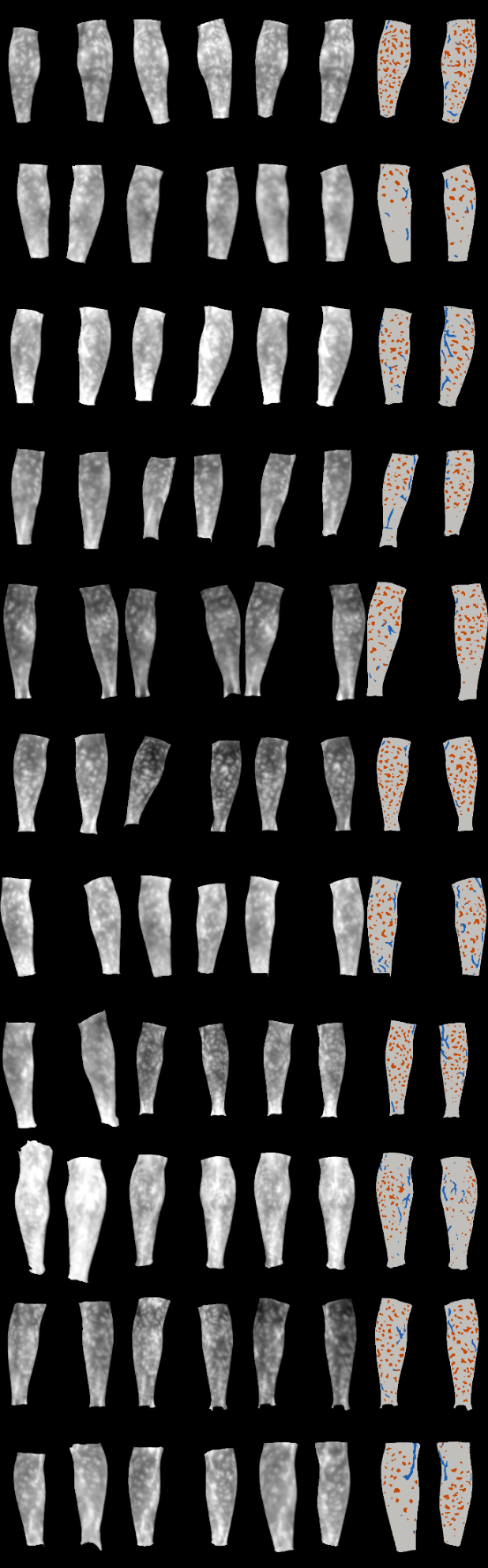


**Supplemental Figure S5.** Core temperature, rate of perceived exertion and non-vessel calf surface temperature responses to repeated treadmill running sessions.

**
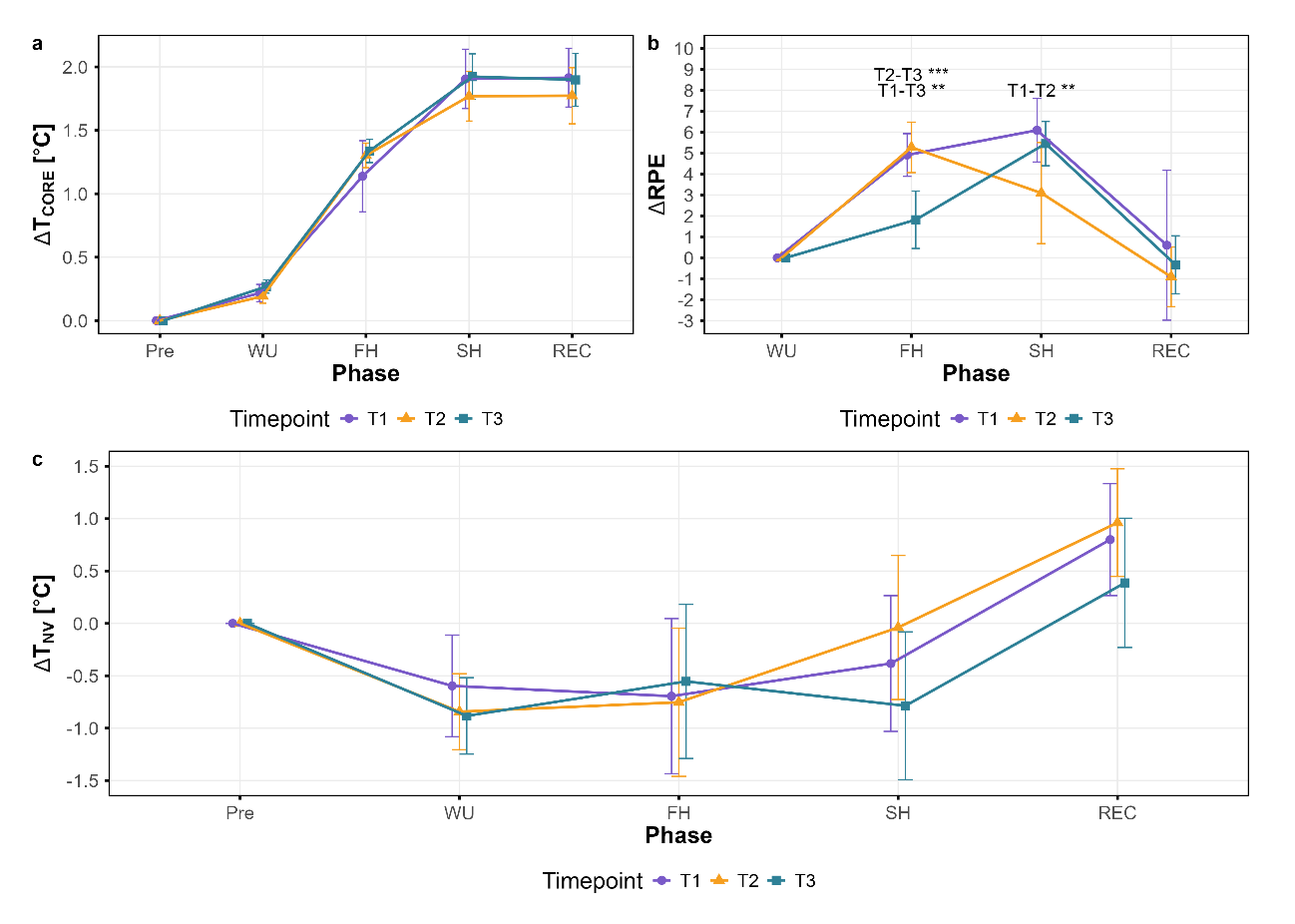
**
